# Long‐term maintenance and effects of exercise in early psychosis

**DOI:** 10.1111/eip.12365

**Published:** 2016-09-01

**Authors:** Joseph Firth, Rebekah Carney, Paul French, Rebecca Elliott, Jack Cotter, Alison R. Yung

**Affiliations:** ^1^ Institute of Brain, Behaviour and Mental Health University of Manchester Manchester UK; ^2^ Greater Manchester West Mental Health NHS Foundation Trust Manchester UK; ^3^ Department of Psychological Sciences The University of Liverpool Liverpool UK; ^4^ School of Psychological Sciences University of Manchester Manchester UK

**Keywords:** early psychosis, exercise, first‐episode, physical activity, physical health

## Abstract

**Aim:**

The aims of this study were to examine if people with first‐episode psychosis (FEP) are able to continue adhering to exercise after a supervised intervention and to explore if the benefits of exercise can be sustained.

**Methods:**

Twenty‐eight persons with FEP took part in a 10‐week exercise intervention that provided each participant with twice‐weekly accompaniment to exercise activities of their own choice, of whom 20 were re‐assessed 6 months after the intervention. Long‐term adherence to exercise was assessed, and measures of psychiatric symptoms, physical health, neurocognition and social functioning were administered at baseline, post‐intervention and 6‐month follow‐up.

**Results:**

During the supervised intervention, participants achieved 124.4 min of moderate‐to‐vigorous exercise per week. After 6 months, physical activity levels had decreased significantly (P = 0.025) and only 55% of participants had continued to exercise weekly. Repeated‐measures analysis of variance found that the significant improvements in psychiatric symptoms and social functioning observed immediately after the intervention were maintained at 6 months (P = 0.001). However, post hoc analyses showed that symptomatic reductions were only maintained for those who continued to exercise, whereas symptom scores increased among those who had ceased exercising. Previously observed improvements in waist circumference and verbal memory were lost by 6 months.

**Conclusion:**

Long‐term exercise participation is associated with significant benefits for symptoms, cognition and social functioning in FEP. However, adherence to unsupervised exercise is low. Future research should explore the effectiveness of ‘step‐down’ support following supervised interventions, and aim to establish sustainable methods for maintaining regular exercise in order to facilitate functional recovery and maintain physical health.

## INTRODUCTION

Physical inactivity is ranked among the top five causes of mortality worldwide^1^ and is one of the greatest public health problems in developed countries^2^ as it increases the risk of non‐communicable diseases such as diabetes, cancer and cardiovascular disease.^3^ People with schizophrenia are more inactive than the general population,^4^ which heightens their risk of cardiometabolic diseases.^5^ Exercise interventions may be one method for improving physical health and reducing the 15‐year mortality gap observed in this population.^6^

Exercise can also reduce the negative symptoms and cognitive deficits of schizophrenia.^7^, ^8^ These features of illness are often unresponsive to antipsychotic treatment, and yet are detrimental to functional recovery.^9^, ^10^ Therefore, developing effective methods for increasing physical activity in schizophrenia could significantly improve both physical and functional outcomes.^6^, ^7^

The first‐episode of psychosis (FEP) may be the optimal time for implementing exercise for several reasons. First, this is a ‘critical period’ for attenuating the metabolic side‐effects of antipsychotic treatment,^11^ as prevention of cardiometabolic abnormalities may be more feasible than reversing them.^6^, ^12^ Second, persons with FEP are younger and more active than those with long‐term schizophrenia,^13^, ^14^ and thus potentially more readily engaged in moderate‐to‐vigorous exercise. Third, providing effective treatment for negative and cognitive symptoms from the early stages of psychosis reduces the likelihood of enduring disability.^15^, ^16^

In our recent trial of individualized exercise for persons with FEP (‘IBEEP’), participants achieved high levels of weekly moderate‐to‐vigorous physical activity.^17^ This, in turn, was associated with significant improvements in physical health, negative symptoms, cognitive performance and social functioning after 10 weeks. Several other trials have recently shown that supervised exercise in FEP can significantly increase physical activity and fitness, while attenuating the typically observed trajectory of weight gain.^18^, ^19^, ^20^ Furthermore, these pilot studies have observed large effect‐sizes of exercise on cognition and real‐world functioning.^20^, ^21^

However, all of these studies were short‐term interventions which provided supervised exercise sessions to participants. None has examined if exercise can be maintained after the intervention period, and how this may be associated with sustained benefits. Thus, we conducted a follow‐up study to the IBEEP trial, 6 months after the intervention had ended. The aims were to examine long‐term exercise adherence among people with FEP and to explore if the improvements in physical and mental health observed from the supervised intervention were maintained after 6 months.

## METHODS

### Setting and participants

The study was conducted as a 6‐month follow‐up to the IBEEP exercise trial (ISRCTN09150095), which was approved by the North West Research Ethics Committee on 18 December 2013 (REC# 13/NW/0784). A full report of the 10‐week trial is available elsewhere.^17^

The initial IBEEP study recruited persons with FEP from Early Intervention in Psychosis (EIP) services in Greater Manchester, UK. Inclusion criteria were: (i) being a current service user of an EIP service, (ii) age 18–35 years and (iii) experiencing psychological difficulties, defined as having either a score of ≥21 on the Beck Depression Inventory 2.0^22^ or ≥2 on the World Health Organization Disability Assessment Schedule 2.0.^23^ Exclusion criteria were: (i) insufficient English language to complete assessments, (ii) inability to provide informed consent, (iii) pregnancy or (iv) physical health issues that contraindicated exercise, including diagnosed heart conditions, poorly controlled asthma and untreated hypertension. A total of 31 EIP service users were initially enrolled in the IBEEP study, of whom 28 commenced the exercise intervention and 25 were retained over 10 weeks.

### Intervention

IBEEP was conducted as a non‐randomized feasibility study, with all eligible participants been allocated to the intervention group. The exercise intervention is detailed elsewhere.^17^ In brief, participants were provided with 10 weeks of individualized exercise training, tailored to their preferences and needs in order to maximize adherence. While allowing for flexibility around exercise type, each plan was designed to achieve ≥90 min of moderate‐to‐vigorous exercise per week, which is congruent with NHS exercise guidelines and may improve mental health in psychotic disorders.^7^, ^24^

During the 10‐week intervention, three forms of support were offered, including: (i) memberships to community leisure services; (ii) financial re‐imbursement for costs incurred by exercise activities and associated travel and (iii) twice‐weekly accompaniment to exercise by research assistants (who recorded attendance, intensity and duration of sessions). The average amount of exercise achieved by participants was 107 min per week for 10 weeks (thus exceeding the set targets), mostly through supervised gym sessions.

### Procedure – 6‐month follow‐up

After completing the 10‐week intervention, participants still had access to the community exercise facilities from the annual memberships which were set up at the beginning of the intervention. Participants were also provided with brief verbal encouragement to continue exercising from the research assistant who supervised their final session, as they informed the participant that carrying on exercising in future would ‘be good for them’ and would ‘help them to stay healthy’. However, accompaniment to exercise training and financial re‐imbursement for transport costs were no longer available after the initial 10 weeks.

Participants were asked if they would be willing to be contacted 6 months after their intervention to participate in a follow‐up study. All who provided written consent were contacted 6 months after their post‐intervention assessments, in order to examine their long‐term adherence, and to re‐assess their psychiatric symptoms, functional disability, physical health and neurocognitive functioning.

### Adherence outcomes

The primary outcome was number of participants who had continued to exercise by the 6‐month follow up; defined as at least one session of moderate‐to‐vigorous exercise each week. This was measured through a self‐report questionnaire, administered in‐person by a research assistant. The questionnaire was adapted from the ‘exercise formulation questionnaire’ used to plan participants’ individualized programmes when first entering the study,^17^ and also assessed the types of exercise undertaken, desired outcomes among participants and their required support.

The ‘International Physical Activity Questionnaire’ (IPAQ)^25^ was administered at baseline, post‐intervention and 6 months to examine changes in total physical activity, calculated as ‘metabolic equivalent task (MET) minutes’. This score represents the total time engaged in light, moderate and vigorous activities during the last week, quantified in aggregate as multiples of ‘resting metabolic rate’. For instance, walking is scored as 3.3 MET minutes, and so a 10‐min walk each day would add 231 MET minutes to the weekly total scores, whereas 1 hour per week of cardiovascular gym‐training (which scores 8 MET minutes) would add a further 480 MET minutes.^25^

Additionally, the ‘Behavioural Regulation in Exercise Questionnaire 2’ (BREQ‐2)^26^ was used to examine motivations towards exercise in the context of Self‐Determination Theory.^27^ A previous large‐scale validation of the BREQ‐2 in schizophrenia has shown that combining items for ‘identified regulation’ (i.e. personal identification with the benefits of exercising) with items for ‘intrinsic regulation’ (i.e. finding exercise enjoyable without external rewards) provides a valid index of ‘autonomous motivation’, which reliably predicts physical activity levels among people with psychosis.^28^ Therefore, we used this score to examine the association between autonomous motivation and total physical activity in FEP.

### Assessments of mental and physical health

A secondary aim was to explore if beneficial changes observed after 10 weeks of exercise^17^ were maintained 6 months later. To limit the number of statistical comparisons, only measures of mental and physical health which previously showed significant improvement (defined as *P* < 0.05) at 10 weeks were selected for analysis at the 6‐month follow‐up.

The principal change measure was psychiatric symptoms. This was measured using the Positive and Negative Syndrome Scale (PANSS); a structured clinical interview administered by trained research assistants. Psychosocial functioning was assessed using the Social and Occupational Functioning Scale (SOFAS). Participants’ waist circumference was measured using standard procedures. Cognitive testing was administered using the Cambridge Neuropsychological Test Automated Battery (CANTAB) interface,^29^ with several additional tasks generated using ‘PEBL’ (Psychology Experiment Building Language).^30^ The cognitive domains examined (and their respective tasks) are detailed in Table 1.

**Table 1:** Neurocognitive assessments

Cognitive domain	Task(s)	Software
Verbal short‐term memory	12‐word verbal recall	CANTAB
Processing speed	Trail Making Task A	PEBL
	Trail Making Task B	PEBL
Executive functioning	Stockings of Cambridge	CANTAB
Inhibitory control	Erikson Flanker Task	PEBL
Social cognition	Mind in the Eyes	Flash

CANTAB, Cambridge Neuropsychological Test Automated Battery; PEBL, Psychology Experiment Building Language.

### Data analysis

Statistical analyses were conducted in SPSS 20.^31^ Exercise adherence was summarized using sample means and percentages. The association between ‘autonomous motivation’ and physical activity levels (IPAQ total MET minutes) was assessed using Spearman's rank.

Repeated‐measures analyses of variance (anovas) were used to examine changes in outcome measures overtime. Values were checked for normality using Shapiro–Wilk tests and normal probability plots. Sphericity was assessed using Mauchly's *W*, and where assumptions of sphericity were violated, a Greenhouse–Geisser adjustment was applied. Where significant main effects were observed, pairwise comparisons were conducted to examine differences between time points. Due to the exploratory nature of these analyses, *α* was set at 0.05 throughout.

## RESULTS

### Long‐term maintenance of exercise

**Figure 1:**
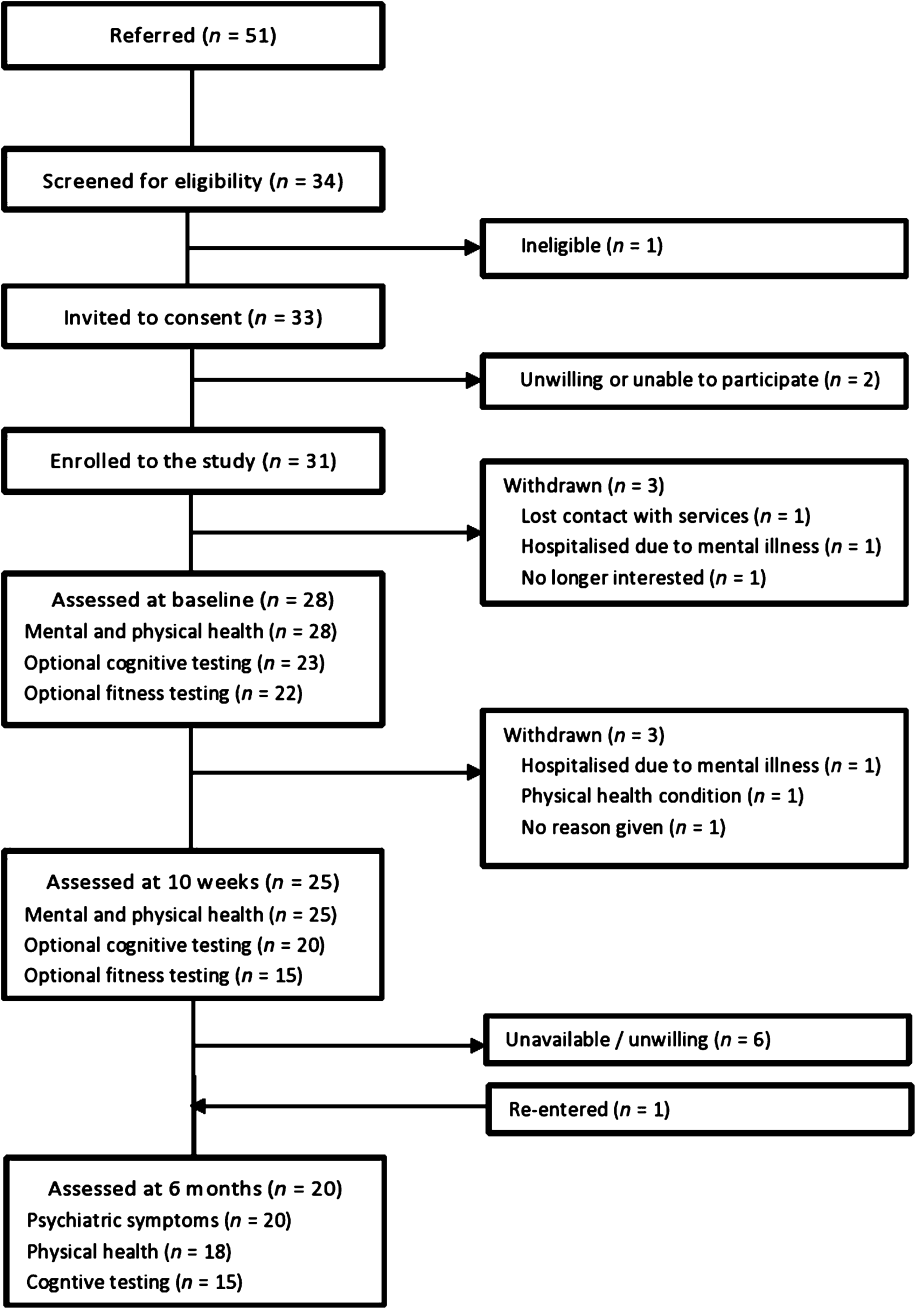
CONSORT diagram of study progress.

Participant progress through the study is displayed in Figure 1. Of the 28 participants in the original IBEEP study, 27 were successfully contacted by telephone 6 months after the intervention. Twenty consented to the follow‐up assessments, and were assessed within 2 weeks of contact (mean = 27 weeks after post‐intervention assessments). Participants had a mean age of 26.96 years (standard deviation (SD) = 4.5 years), were 90% males and had an average EIP service use of 2.49 years. Independent samples *t*‐tests found no significant differences between those who took part in the follow‐up (*n* = 20) and those who did not (*n* = 8) in baseline psychiatric symptoms, waist circumference, body mass index (BMI) or social functioning. However, participants in the follow‐up study had achieved significantly more moderate‐to‐vigorous exercise during their 10‐week intervention (124.4 min per week) in comparison to those who opted out of the follow‐up (62.4 min per week, *P* = 0.013).

The adherence questionnaire showed that 11 of 20 participants had continued to exercise weekly, whereas 9 of 20 participants exercised less than once per‐week or not at all. The most common types of exercise activities were gym sessions (60%), walking/hiking (40%), home exercises (e.g. callisthenics, free‐weights or aerobics videos) (40%) and cycling (20%). The types of activities people wanted to do more of were gym training (70%), jogging (35%), cycling (25%) and swimming (20%). Participants were also asked which of the three types of possible support would help them to exercise more. The most popular option was ‘training partner’, endorsed by 75% of participants. ‘Transport to exercise location’ was selected by 40% and ‘more exercise advice’ by only 15%.

Participants’ scores for ‘autonomous regulation’ (calculated from the BREQ‐2) held moderately strong positive correlations with IPAQ total MET minutes at the 6‐month follow‐up (*r*_s_ = 0.471, *P* = 0.042), indicating that maintenance of physical activity was significantly associated with participants’ enjoyment of it and personal identification with the benefits.

### Changes in physical and mental health

The results of all repeated‐measures anovas are displayed in Table 2. Notable findings are detailed below, using summary statistics to describe the magnitude of changes from baseline (T1), to immediately following the exercise intervention (T2), to the 6‐month follow‐up (T3).

**Table 2:** Changes in outcome measures 6 months after the exercise intervention

	Repeated‐measures anova			Mean scores (SD)			Pairwise comparisons (*P*‐value)		
	*F*‐value	Total *n*	*P*‐value	Baseline T1	Post‐exercise T2	6 months later T3	T1 to T2	T2 to T3	T1 to T3
PANSS total	10.18	19	<0.001	77.7 (16)	67.2 (12)	65.9 (14)	0.002	0.647	0.001
Positive symptoms†	3.89	19	0.042	19.3 (6.0)	16.8 (5.6)	17.5 (6.0)	0.001	0.490	0.1180
Negative symptoms	7.46	19	0.002	18.7 (5.0)	15.1 (4.8)	14.7 (3.6)	0.007	0.522	0.003
General symptoms	6.53	19	0.004	39.7 (7.6)	35.0 (5.4)	33.8 (7.5)	0.024	0.462	0.002
SOFAS	9.37	19	0.001	46.6 (7.8)	50.2 (8.6)	55.6 (10)	0.039	0.025	0.001
IPAQ total MET minutes†	4.15	17	0.042	759 (744)	1929 (1147)	1362 (1834)	0.001	0.290	0.129
Moderate/vigorous MET minutes	25.27	17	<0.001	250 (118)	1237 (229)	355 (109)	<0.001	<0.001	0.320
Sitting time (min per day)	3.03	11	0.069	385 (51.4)	272 (34.9)	290 (26.5)	0.093	0.658	0.059
Waist circumference (cm)	7.25	17	0.003	105.6 (16.6)	103.2 (17.3)	107.2 (18.4)	0.008	0.003	0.212
Verbal STM	4.47	13	0.022	7.1 (1.6)	8.4 (1.9)	6.6 (2.5)	0.012	0.023	0.513
Trail Making Task A (s)	4.57	14	0.020	24.5 (7.3)	20.4 (4.7)	20.2 (4.8)	0.002	0.888	0.041
Trail Making Task B (s)†	9.21	14	0.004	36.3 (9.1)	29.8 (9.3)	27.9 (7.7)	0.001	0.468	0.001
Eyes task	1.78	12	0.192	19.4 (5.4)	20.8 (6.7)	19.4 (6.3)			
Stockings of Cambridge	3.08	13	0.065	6.9 (2.1)	7.9 (2.1)	8.0 (1.9)			
Flanker conflict error %	2.87	10	0.083	27.8 (27)	10.1 (8.4)	18.0 (22)			

† Greenhouse–Geissure adjustment applied for sphericity violation.

anova, analysis of variance; IPAQ, International Physical Activity Questionnaire; MET, metabolic equivalent task; PANSS, Positive and Negative Syndrome Scale; SD, standard deviation; SOFAS, Social and Occupational Functioning Scale.

Significant main effects were observed for PANSS total scores and also for each subscale. Pairwise comparisons between time points found significant improvements after exercise (i.e. between baseline and T2) in total symptoms, positive symptoms, negative symptoms and general symptoms. Significant improvements from baseline to T3 were found for PANSS total scores (−24.6% from baseline, *P* = 0.001), negative symptoms (−34.2%, *P* = 0.003) and general symptoms (−24.9%, *P* = 0.002). Changes in positive symptoms from baseline to T3 were not significant (−14.6%, *P* = 0.118). Neither PANSS total scores nor subscales showed any significant changes between intervention completion and follow‐up (all T2 to T3 comparisons *P* > 0.05).

Social functioning was the only outcome to show continued improvements after the intervention had ended; increasing by 7.8% from baseline to T2 (+3.63 SOFAS points, *P* = 0.039), and then a further 10.7% from T2 to T3 (+5.37 SOFAS points, *P* = 0.025). Total physical activity levels at T2 were 2.5 times higher than baseline (*P* = 0.001, even after removing one high‐exercising outlier). However, significant increases in both total physical activity and moderate‐to‐vigorous activity from baseline were lost by 6 months, while there were no significant changes in sedentary behaviour overtime (Table 2). Waist circumference was also significantly reduced after the intervention (T1 to T2, 2.4 cm, *P* = 0.008), although participants gained 4 cm between T2 and T3 (*P* = 0.033), leaving final measurements no different from baseline (T1 to T3, *P* = 0.212).

In cognitive testing, improvements in Trail Making Tasks A and B were maintained at the 6‐month follow up (T1 to T3, *P* = 0.042 and *P* = 0.001, respectively). Verbal short‐term memory increased significantly from T1 to T2 (+18.3%, *P* = 0.012), followed by significant worsening between T2 and T3 (−21.4%, *P* = 0.023), thus returning to baseline performance. No main effects were found in the Flanker task, Stockings of Cambridge or Reading the Mind in the Eyes.

**Figure 2:**
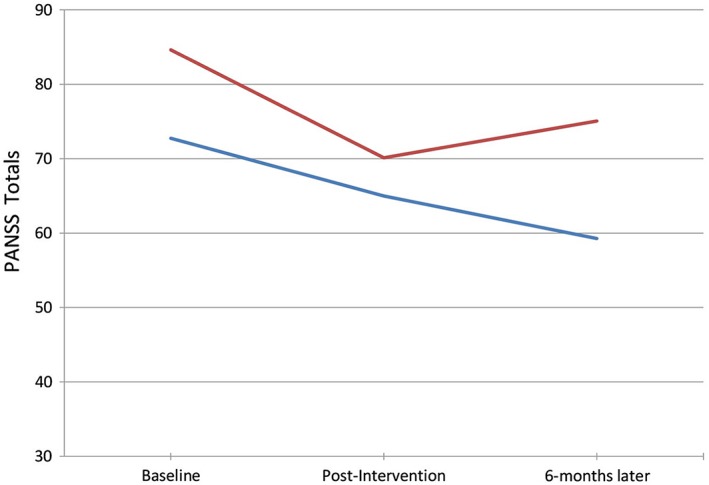
Changes in psychiatric symptoms (Positive and Negative Syndrome Scale (PANSS) total scores) in those who continued to exercise versus those who ceased exercise after the supervised intervention (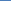, exercisers (n = 11); 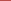, non‐exercisers (n = 8).

To explore the association between exercise adherence and long term‐benefits, a post hoc *t*‐test was conducted to compare changes in PANSS total scores after 6 months in participants who continued to exercise on a weekly basis (*n* = 11) to those who did not (*n* = 8). There was a significant difference between the two groups (*t* = −2.18, *P* = 0.044), as weekly exercisers continued to show symptomatic reductions 6 months after the intervention (T2 to T3; −16.4% PANSS total scores) while non‐exercisers’ symptoms increased after the intervention period (T2 to T3; +12.3% PANSS total scores). Changes in symptoms over the course of the intervention and follow‐up period are shown in Figure 2.

## DISCUSSION

This was the first study to examine the long‐term adherence and associated benefits of exercise in FEP. A total of 20 young adults with FEP took part in follow‐up assessments 6 months after receiving 10 weeks of individualized exercise training. This study found that sustained support may be required to maintain exercise following a supervised intervention: Although participants still had access to exercise facilities, only 55% had continued to exercise after 6 months. This was despite the high levels of physical activity achieved during the supervised period. These findings are consistent with previous research in long‐term schizophrenia, which shows that while supervised exercise results in high levels of exercise adherence and related health benefits, providing exercise access and advice without social support does not.^32^, ^33^

The importance of maintaining exercise over the long‐term is supported by the observed changes in psychiatric symptoms. PANSS total scores were significantly reduced after 10 weeks of exercise, particularly for negative symptoms.^17^ Sustained symptomatic improvements after 6 months were also associated with continued adherence to exercise. As shown in Figure 2, those who had ceased exercising after the intervention had lost the symptomatic benefits by the follow‐up point, whereas those who exercised on a weekly basis continued to show significant improvements after 6 months.

We found significant increases in verbal Short Term Memory (STM) during the supervised exercise period, a domain of cognitive functioning which has proven sensitive to exercise in psychosis.^17^, ^34^ However, verbal STM had reverted to baseline scores by 6 months after the intervention. These findings are consistent with research in the general population showing that exercise‐induced increases in verbal memory only persist when regular physical activity is maintained.^35^ There is also increasing evidence of a dose–response relationship between exercise and neurocognitive performance in persons with schizophrenia.^8^, ^17^, ^36^ The early stages of psychosis could be the ideal time frame for using exercise to improve long‐term cognitive outcomes, since cognitive enhancement interventions are more effective at this time,^15^ perhaps due to an increased capacity for neuroplasticity in younger people.

Early psychosis is also a critical period for physical health outcomes.^6^, ^11^, ^16^ In this study, 10 weeks of supervised exercise resulted in a 150% increase in physical activity and a 2‐cm reduction in waist circumference (*P* = 0.001 and *P* = 0.008, respectively). However, by 6 months after the intervention, physical activity levels were on a downward trajectory and waist circumference had increased by 4 cm. As both physical activity and waist circumference are stronger predictors of cardiovascular risk than body weight/BMI,^37^, ^38^ sustainable exercise programmes should be offered from the initiation of antipsychotic treatment to attenuate the decline in metabolic health and associated mortality.

Thus, our findings suggest that some form of long‐term exercise facilitation would have a positive impact on symptomatic, functional and physical health outcomes among young people with psychosis. In our sample, 75% of participants felt that having someone to train with would help them to exercise more. A recent meta‐analysis of survey data has also found that ‘having no one to train with’ is one of the most common modifiable barriers towards exercise among those with psychosis.^39^ Novel approaches towards providing social support after a supervised intervention has ended should be explored. Potentially feasible methods to achieve this include pairing‐up participants after the intervention as training partners, enabling service users to become peer coaches, using smartphone apps to provide remote coaching and exercise tracking after an intervention or employing exercise professionals to run regular group training sessions for mental health services.

Another factor relevant to long‐term exercise engagement is the role of autonomous motivation from self‐determination theory.^27^ Previous cross‐sectional studies have shown that levels of autonomous motivation among people with psychosis predict their total physical activity levels.^28^ Even in this small sample, a significant correlation was found between autonomous motivation and IPAQ physical activity scores 6 months after the intervention (*r*_s_ = 0.471, *P* = 0.042), indicating that participant's enjoyment of exercise, and their personal identification with the benefits, was associated with greater adherence overtime.^28^ According to Ryan and Deci,^27^ autonomous motivation results from (i) the freedom experienced when engaging in preferred forms of exercise (‘autonomy’); (ii) the ability to achieve desired outcomes through exercise (‘competence’) and (iii) being socially connected (‘relatedness’). Designing interventions to support these motivating factors could boost long‐term adherence and reduce the need for external incentives or intensive support.

### Limitations and future research

One limitation of this study is the small sample size. An attrition rate of 29% by the 6‐month follow‐up also introduces the possibility of a ‘survival bias’ influencing our findings. This is further indicated by the significant differences in exercise engagement observed between those who took part in the follow‐up and those who did not.

Another limitation is that although significant differences in psychiatric symptoms were found between exercisers and non‐exercisers at 6 months, the directionality of these effects cannot be fully established by this observational study. For instance, it is possible that patients’ whose symptoms are responding well to treatment are capable of continuing to exercise without supervised interventions, while those who were experiencing a decline in mental health may be unable to exercise unassisted. It should also be considered that exercise engagement at the 6‐month follow‐up was assessed using a self‐report questionnaire. This introduces a further limitation to the study, as subjective measures typically overestimate physical activity in comparison to gold standard objective measures such as accelerometry.^4^

Furthermore, as exercise engagement at the 6‐month follow‐up was assessed using a self‐report questionnaire, this can also be considered a limitation surrounding subjective measures of physical activity should also be considered, as subjective measures of physical activity differ significantly from objective‐measured physical activity among persons with psychosis.

A large‐scale randomized trial of exercise in FEP using intention‐to‐treat analyses over an adequate follow‐up period could overcome these limitations, and thus determine the lasting effects of exercise on physical and mental health outcomes in FEP. Future interventions should also aim to facilitate long‐term adherence to regular moderate‐to‐vigorous exercise. This could involve a brief introductory period of relatively intensive exercise supervision, followed by stepped‐down support in the form of peer‐to‐peer coaching, internet‐enabled trainer contact or referral to community‐based exercise groups. Such interventions should foster feelings of autonomy, competence and relatedness to embed physical activity within the daily lives of young people with FEP, in order to prevent physical health decline and improve functional recovery.
